# Use of biochar from rice husk pyrolysis: assessment of reactivity in lime pastes

**DOI:** 10.1016/j.heliyon.2021.e08423

**Published:** 2021-11-18

**Authors:** Luisa F. Morales, Katherine Herrera, Julián E. López, Juan F. Saldarriaga

**Affiliations:** a Dept. of Civil and Environmental Engineering, Universidad de Los Andes, 1Este #19A-40, 111711, Bogotá, Colombia; b Environmental Engineering Program, Universidad de Medellín, Carrera 87 #30-65, 050026, Medellín, Colombia

**Keywords:** Rice husk, Pyrolysis, Biochar, Reactivity, Lime pastes

## Abstract

Biochar has unique properties such as its porous structure, specific surface area, and stable chemical properties. The rice husk is characterized by its high content of silica, and that during the pyrolysis process it generates a considerable amount of biochar that can be used in different processes. The aim of this work is to evaluate several biochars from the pyrolysis process in the reactivity of lime pastes. For this, biochar has been obtained at four different temperatures (450, 500, 550 and 600 °C), and they have been characterized by XRF, XRD, ICP-EOS, and particle size distribution, to determine their phases and their chemical composition. Biochar has been replaced in lime pastes in different proportions (5, 10, 15, 20, 25 and 30%), and exposed to different curing times (1, 3, 7, 14, 28, 56, 90 and 180 days). It has been found that all the replacements show reactivity within the lime pastes and that the percentage of 25% in all the biochar tested could be an adequate replacement.

## 1 Introduction

Biochar has unique properties such as porous structure, large specific surface area, complex surface groups, and stable chemical properties [1, 2]. Biochar comes from the pyrolysis process, in which the volatile matter, constituted mainly by hemicellulose, cellulose, and polysaccharides, is mostly eliminated, and its degradation depends on the temperatures of the process [3, 4, 5, 6, 7, 8]; making the biochar mainly composed of lignin. Biochar has been found to be the only one subproduct among the thermal technologies that can provide the potential to address some problems that happen in the cement industry, such as the reduction of CO_2_ emissions in the world [9]. This is considered adequate because it does not present impurities in the ash content, one of the main problems that has been found with the rice husk in the substitution of mortars [10]. Another problem that biochar can solve as a replacement is that it does not present hemicellulose contents as sources of sugars, including glucose and fructose, which delay hydration processes [11].

Biochar has been widely used to develop construction materials such as geopolymers [9, 12, 13], natural inorganic clay compounds [14], bituminous material [15, 16], red clay binders [17], among others. Some authors argue that when biochar is added to building materials, carbon can be stored and locked in the structure for decades, helping to keep global carbon cycles in balance, due to the sequestering nature of biochar [9]. Currently, by-products of biological origin are used that have not shown as many advantages as the use of biochar from the pyrolysis process, making biochar a good replacement for cement in the production process in industry. With the use of biochar, it is possible to reduce up to 870 kg of CO_2_ equivalent of greenhouse gases (GHG) per ton of dry raw material [18, 19].

The use of this type of material is not new and, in the cement industry, it has been carried out for several decades, due to its pozzolanic, morphological, and micro-aggregated effects, which can be used to produce concrete with adequate properties and worthy yields [20, 21]. Furthermore, the benefits of using this type of materials are better workability, reduced bleeding and less water demand, improved resistance to corrosion of reinforcing bars, reduced drying shrinkage, better resistance to alkali-silica reactivity, reduced carbon footprint in the concrete industry, etc. [20, 21, 22, 23, 24, 25, 26].

The type of raw material from which the biochar comes, and the burning temperatures affect the suitability of use in cementitious binder systems. However, the most widely used by-product in the cement industry is fly ash, which is traditionally used in a range of 5–50% by mass per Portland cement [27, 28, 29, 30], although more recent studies have shown that even 60% replacements with fly ash are adequate [31, 32]. Nevertheless, some authors argue that it is still necessary to perform laboratory tests before the use of any particular mix design, because the behavior and performance of cements based on Portland cement is not as predictable [33]. Not all types of fly ash and biochar can be used as a substitute for cement, since some must be activated by chemical methods (borax or NaOH) or by physical methods; others can just be activated and some cannot even be used directly with concrete [34, 35, 36, 37, 38]. These differences then prevent a more extensive use of these materials, especially hundreds of millions of tons of fly ash and biochar that do not comply with current regulations [39, 40] and that are currently being deposited in open dumps, especially in developing countries such as Colombia.

The replacement of cement by biochar/fly ash depends on the content of silica and its chemical reaction with the cement compounds. The reactions between siliceous or silico-aluminous material that chemically reacts with calcium hydroxide at ordinary temperatures forms compounds that have cementitious properties [41]. The composition of the biochar and the fly ash, favors the hydration of the calcium silicate compounds, C_3_S (or alite) and C_2_S (or belite), and this can vary according to the composition of the source materials. These phases are generally represented simply as the C–S–H gel, which indicates that it does not have a rigorous stoichiometric composition. C–S–H gel is the primary cementitious compound in Portland concrete and is largely responsible for providing strength and other desirable properties to concrete [41].

C–S–H gels that are formed as a result of the pozzolanic reaction of silica and alumina of pozzolana, with the calcium hydroxide produced by the hydration of Portland cement, is one of the main mechanisms behind the technical benefits attributed to the use of pozzolans with Portland cement in concrete [41]. The addition of fly ash/biochar influences many parameters like fresh concrete properties such as workability, rheology, air content, setting behavior, hydration rate and heat release, pore solution chemistry evaluation, microstructure, mechanical properties, volume stability, durability, among others [41].

In recent decades, there has been a growing demand for the use of this type of materials, which although they do not meet standards, these can be recycled in the concrete industry [20]. Some authors estimate that the infrastructure construction industry will have a growing demand for fly ash and it is expected to exceed 35 million tons by 2030, although it is projected that the supply will be only 14 million tons [20, 42]. This enormous gap between supply and demand requires not only the recycling of fly ash that is being improperly disposed but also the use of biochar [20]. This is why the reactivity evaluation is an important parameter to determine if this material has the capacity to be pozzolanic or cementitious, that is, the ability to activate, depolymerize, hydrate and repolymerize to generate hydration products [20]. At present, no single index (or combined indices) has yet been established to characterize the reactivity of fly ash and biochar fully and reliably. TGA analyses have been widely used to determine the formation of the different crystals and gels inherent to the process, but there is still an urgent need to further explore the reactivity of these materials, including biochar [20, 28, 30].

The aim of this work is to evaluate four types of biochar obtained from the rice husk pyrolysis process at four different burning temperatures, using different replacement percentages as supplementary for the cement. For this, the reactivity of the biochar in lime pastes has been analyzed, based on thermogravimetry as a characterization technique to determine the phases of the cement. With this work it will be possible to find new uses for the biochar from the rice husk pyrolysis, which is currently being widely studied for the removal of pollutants in water and soil. Similarly, novel evidence is presented of the potential use of biochar in the replacement of cement for the manufacture of low-performance inputs. Thus, leading to the creation of new perspectives for the application of biochar in this area as important as construction. Giving it a contribution to the circular economy through the treatment of waste from pyrolysis and the use of a by-product in this industry.

## 2 Experimental

### 2.1 Materials

Four types of biochar have been obtained from a pyrolysis process described in Part A. Table 1 presents the chemical composition of all biochars determined by X-ray fluorescence (XRF) in a ZSX Primus Rigaku spectrometer (Tokyo, Japan).

**Table 1:** Chemical composition of the biochars determined by XRF.

Compound (wt. %)	Biochar			
	450	500	550	600
SiO_2_	89.57	89.45	89.07	89.65
Al_2_O_3_	0.85	0.92	0.99	0.83
Fe_2_O_3_	1.12	1.26	1.38	2.28
CaO	1.57	1.85	2.07	2.02
MgO	0.57	0.57	0.63	0.33
SO_3_	0.09	0.08	0.27	0.14
K_2_O	5.48	5.11	4.73	3.81
Na_2_O	0	0	0.12	0
Ti_2_O_3_	0.21	0.23	0.23	0.26
P_2_O_5_	0.39	0.39	0.36	0.51
Mn_2_O_3_	0.11	0.13	0.14	0.15
LOI	0.04	0.02	0.02	0.03

LOI implies loss on ignition.

A high SiO_2_ content is observed in all biochar, and all can be classified type F according to ASTM-C618 standard [40]. A similar composition is observed in the content of SiO_2_, Al_2_O_3_, and Fe_2_O_3_, which are 91.54, 91.63, 91.44 and 92.72 wt% for the 450, 500, 550 and 600 biochars, respectively. According to the ASTM standard [40], all biochars can be used as a substitute for cement if the percentage of SiO_2_, Al_2_O_3_, and Fe_2_O_3_ represents more than 70% w/w and in all it is observed that this percentage is greater than 90%. The lime used is Corona brand, with a chemical composition of CaO of 93.7%, SiO_2_ content of 2.9%, Fe_2_O_3_ of 1.0% and one of them are ignition of 2.4%.

An ICP-OES analysis has also been performed to quantify the metal concentration using inductively coupled plasma optical emission spectrometry (ICP-OES) in an ICP-OES Thermo Scientific ™ ICAP6500 DUO kit Thermo Scientific equipment (Waltham, USA). It is observed that the only metal present in the sample is barium, the rest of the metals are below the detection limit or between the detection limit and the quantification limit (Table 2). These results agree with those found in the XRF analyses (Table 1).

**Table 2:** Metal content determined by ICP-OES.

Parameter (mg L^−1^)	Biochar			
	450	500	550	600
Silver	<0,035	"0,039″	"0,041″	"0,038″
Arsenic	<0,035	<0,035	<0,035	<0,035
Barium	0.240	0.656	0.648	0.583
Cadmium	<0,035	<0,035	<0,035	<0,035
Chromium	<0,035	0.218	<0,035	<0,035
Mercury	<0,070	<0,070	<0,070	<0,070
Led	<0,035	<0,035	<0,035	<0,035
Selenium	<0,035	<0,035	<0,035	<0,035

<XXX: Value below the detection limit of the method.

“XXX”: Value between the limit of detection and the limit of quantification.

**Figure 1:**
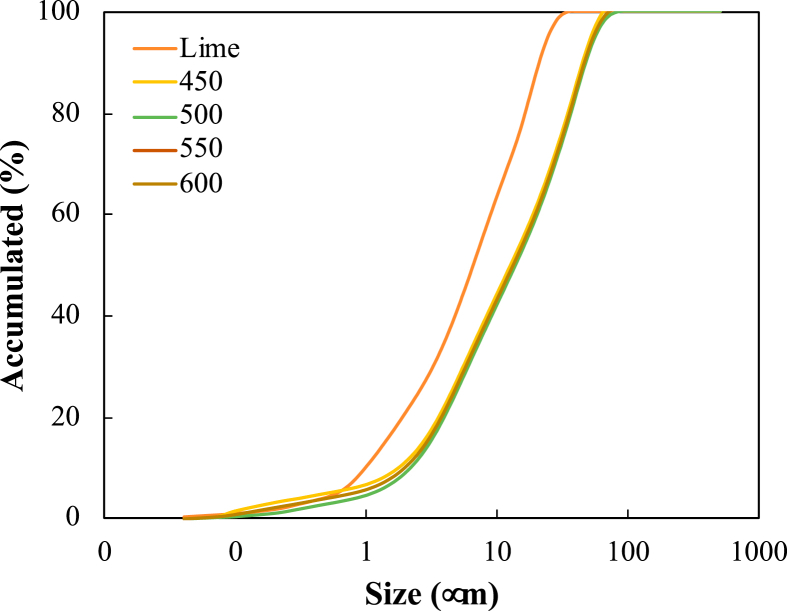
Cumulative distribution of particle sizes for lime and biochar.

The particle size distribution has been determined by laser granulometry. It has been carried out for the four biochars using a CILAS 1064 particle analyzer (Orléans, France) by the laser method, using isopropyl alcohol as dispersing agent, in a range between 0.1 and 500 mm, according to the SM 2560D method of the Standard Methods [43]. Figure 1 shows the accumulated particle distributions for lime and the four biochars. A similarity is seen in the biochars, where their sizes are observed between 1 to 10 μm, a similar behavior obtained by Arenas-Piedrahita et al. [44]. It is then observed that the particle size observed for lime is a little smaller than for biochars, causing a small heterogeneity which could affect the pozzolanic reactions. This means that the crystal formation reactions can be a little slower than those produced by cement alone, leading to the fact that this material can only be used in low-performance inputs and not in structural systems [45].

### 2.2 Preparation of lime pastes

Lime pastes have been prepared in accordance with the ASTM-C305 standard [46]. A water/lime ratio of 0.5 has been selected to guarantee complete hydration of the pastes [28, 30, 47]. The lime substitution has been 5, 10, 15, 20, 25 and 30% w/w for each biochar tested. The mixtures have been identified as XXX-Y, where XXX represents the type of biochar (450, 500, 550 and 600) and Y represents the percentage of substitution. The pastes have been cured in a water bath avoiding water/lime contact in order not to alter the water/lime ratio. The hydration process has been stopped with acetone at the ages of 1, 3, 7, 14, 28 and 56 days, then the samples have been taken to an oven at 60 °C for 1 h to guarantee the complete elimination of the water and acetone content.

### 2.3 Evaluation of the reactivity of lime pastes

Thermogravimetry tests have been developed on a TGA-5500 Discovery from TA Instruments (New Castle, USA). The samples have been heated from 30 to 600 °C with a heating rate of 10 °C min^−1^ and N_2_ UAP purge gas. Similarly, high resolution thermogravimetric analyses have been carried out to observe the increase in pozzolanic reactions. The maximum temperature was established at 250 °C with a heating ramp of 50 °C min^−1^ [48]. Different resolutions and sensitivities have been tested in the TGA, establishing that the best relationship has been a resolution of +5 and a sensitivity of 3, since in these parameters a better separation of the peaks comprised in a temperature between 40 and 200 °C.

### 2.4 Characterization of ashes and pastes

To evaluate the phases, present in the biochar XRD tests have been carried out using a Miniflex-Rigaku X-ray diffractometer (Tokyo, Japan) working on 180 Bragg-Brentano geometry with Cu-Ka 1.2 wavelengths (1.54051 and 1.54433 ºA). The diffractometer has been operated in the angular range of 2θ = 6−80°, using a 0.02° step (2θ) and an acquisition time of 2 s per step. To determine functional groups, FT-IR analysis was used an infrared spectrometer PerkinElmer, model spectrum Two V10.4.2 (Waltham, USA) equipped with an Attenuated Total Reflection (ATR) accessory (PerkinElmer) was employed, operating in the spectral range 4000–400 cm^−1^ with a resolution of 4 cm^−1^.

## 3 Results and discussion

### 3.1 Determination of the phases of biochar

**Figure 2:**
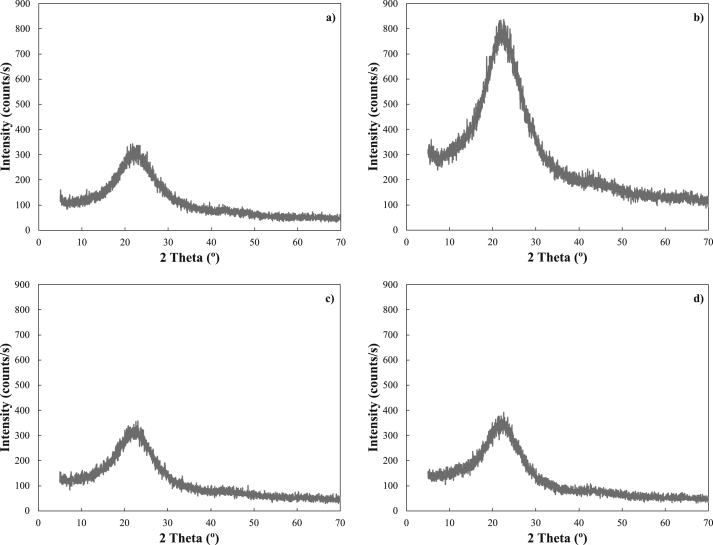
XRD analysis of the four biochars tested in this study, a) 450, b) 500, c) 550, and d) 600.

Figure 2 shows the diffractograms obtained for each of the biochars evaluated in this study. This analysis has been used to determine the amorphous nature of biochar. The XRD patterns show similar materials as expected and indicate a long-range amorphous material. There is a main broad peak in the low angle region (10º-35°) is indexed as (002) stacking of the graphitic basal plan in biochar [49, 50, 51, 52, 53, 54]. It is also observed that the four biochars present a comparable and predominantly amorphous structure, with crystallite fringes assigned to the graphitic phase and that coincide with other works published in the literature [49]. These results demonstrate that biochar influences pozzolanic reactions as well as mechanical performance, since this type of phase permits stability and durability [55, 56]. Although crystalline phases are not observed in the sample, Figure 2b shows a greater intensity with respect to the others, it may be since possibly in the evaluated sample there was a high number of atoms that favored a high number of electrons in the unit examined that could have caused this effect. But as mentioned, these samples are totally amorphous, which does not present a problem for the experiments, since there are no crystalline phases that can cause problems in their reactivity with lime.

### 3.2 Analysis of the reactivity of lime-biochar pastes

**Figure 3:**
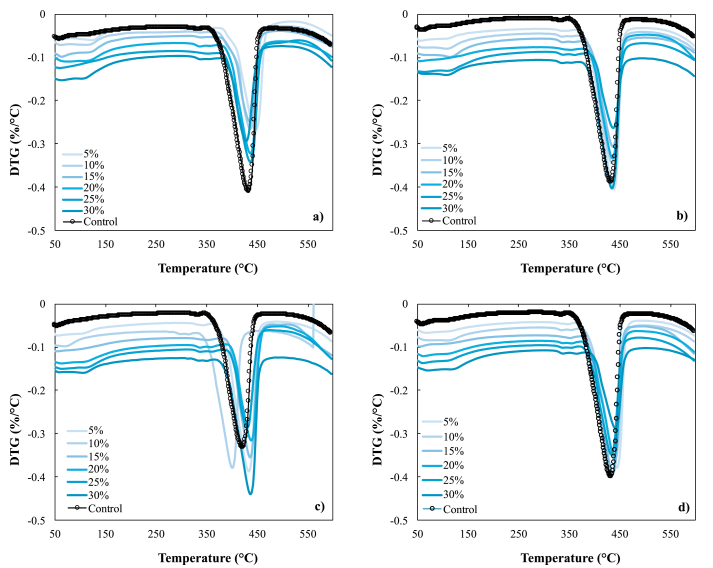
Evolution of the formation of C–S–H and Ca(OH)_2_ gels for different hydration at 56 day, a) 450, b) 500, c) 550 y d) 650.

Figure 3 shows the evolution of the hydration process of the cement pastes for all the biochar tested and the control. It has been observed that, as the hydration time passes, a clear transformation of the portlandite occurs (peak between 350 and 450 °C) to one of the C–H–S gel phases (peak between 50 and 150 °C). The TGA tests have been carried out in triplicate in all the replacements implemented, to observe the typical reactions of formation of C–S–H gels. Minimal differences have been evidenced between all the biochars in the C–S–H gel formation process, this means that despite having carried out pyrolysis at different temperatures, and finding some changes in their chemical composition, the behavior is very similar. Therefore, a comparable behavior is seen with all the replacements, and even in 25% of replacements in the 500 (Figure 3b) and 550 (Figure 3c) is observed a better formation of these gels. Also, when comparing all the biochar tests in contrast with lime as a control, it is demonstrated that the latter does not present significant peaks in the range between 50 and 150 °C. These peaks are clearer as the age of cure increases and it is principally due to the consumption of portlandite [28, 48].

With the results shown in Figure 3, the formation of both C–S–H gels and also portlandite is evidenced, which according to other authors is the ideal behavior in the hydration process, since in this way the resistance to compression is present in any associated material [57].

**Figure 4:**
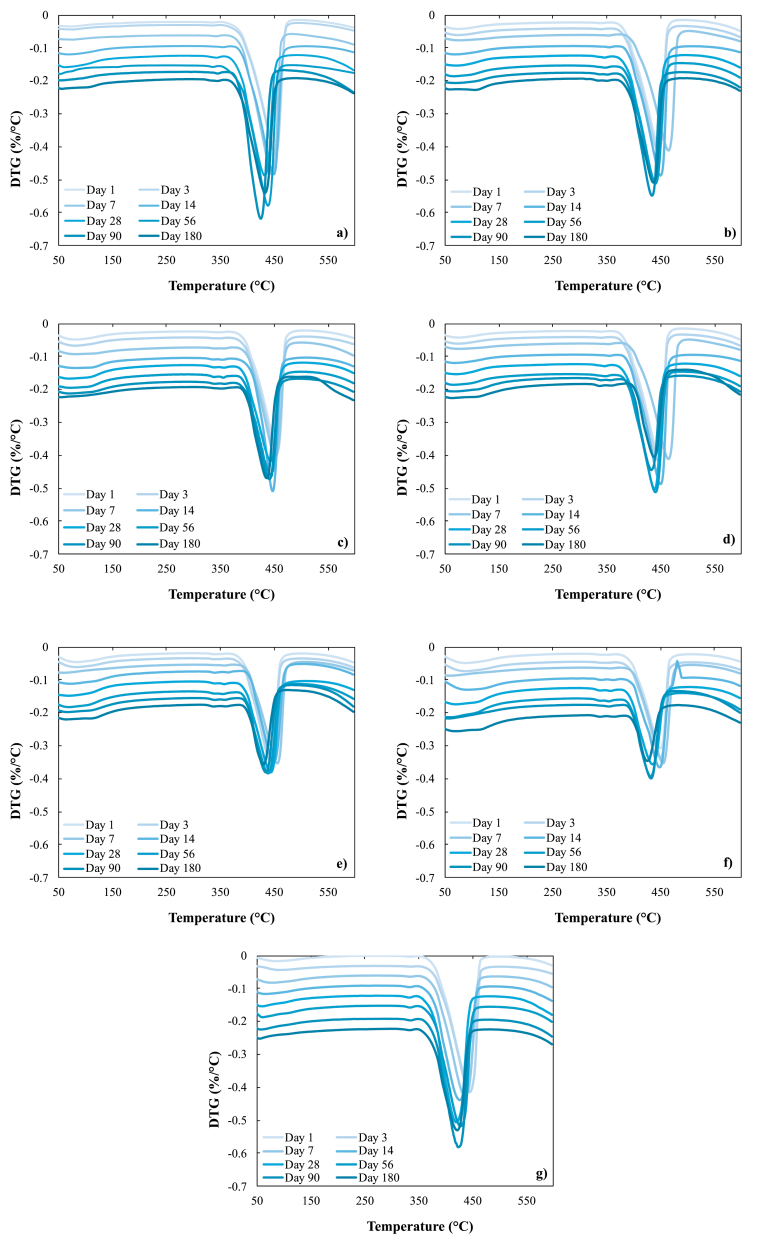
Thermogravimetric analysis for the 550 biochar samples in the different replacements, a) 550–5, b) 550–10, c) 550–15, d) 550–20, e) 550–25, f) 550–30 and g) control.

Figure 4 shows the evolution of the 550-biochar curing process in all the replacements carried out at all times of curing and compared with the control (only lime). It is evident the good behavior of all the replacements carried out and that as the curing time passes, the formation of a peak between 50 and 150 °C is observed. This peak is associated, according to some authors, presumably to C–S–H gels [58, 59] formation (Figure 7), and this behavior is adequate according to the characterization and classification of all the biochars tested in this study (Table 1) as type F.

It is also observed in Figure 4, that on days 14 and 28 there is a decrease in portlandite, that can probably be transforming into some type of C–S–H phase [47, 60]. This behavior has been evidenced in all biochars. Subsequently, on day 56 until day 180 an increase in the most pronounced peak is shown, even for the C–S–H gels and C-A-S-H (Figure 7), indicating in effect that with the use of mineral additions in the concretes, the biochars-lime do not develop resistance at early ages but at late ages [44, 61].

### 3.3 High-resolution thermogravimetry assays

**Figure 5:**
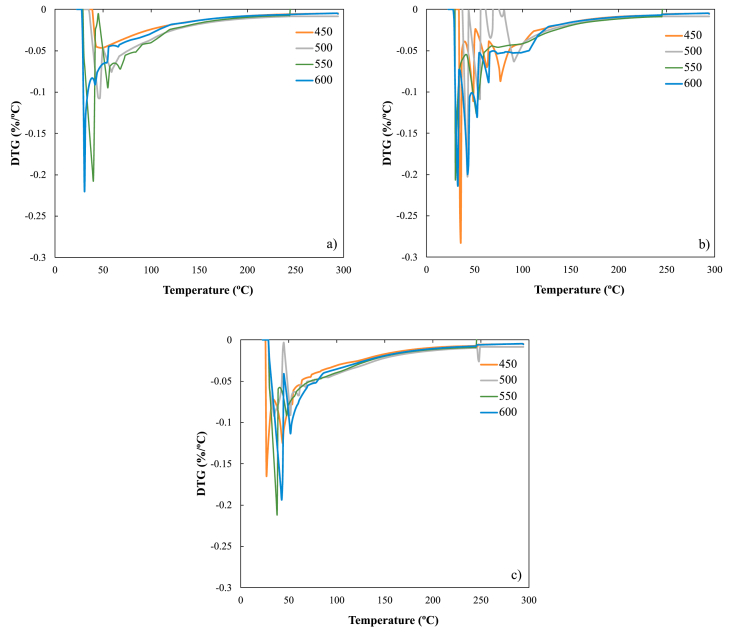
High resolution thermogravimetry of lime-biochar mixtures with a 25% w/w replacement after: a) 56 day, b) 90 day and c) 180 day.

Figure 5 shows the high-resolution analysis, which seeks to evaluate the formation of these C–S–H gels that may be forming in the range between 50 and 150 °C. It is evident that after 56 days of curing, said gels and hydrated calcium aluminosilicates are being formed, according to that reported by Song et al. [62]. Consistent with different authors, hydrated calcium aluminosilicates correspond to gypsite or ettringite, due to the reactions of biochar with portlandite to form the different types of C–S–H [9, 20, 22, 44, 63, 64, 65]. These results can be contrasted with those shown in Figures 4 and 7, in which it is evidenced that there is a reactivity of all the biochars tested in this study. From day 56 a formation of the C–S–H gels is evident (Figure 4a), but on day 180 a better definition of the peaks that are associated with the C–H–S gels is observed, which implies that it is not necessary to verify their existence with high resolution TGA tests. Likewise, it is verified with this work that from the pyrolysis of rice husk, at any temperature a biochar has been obtained that can be used as a replacement for cement; offering adequate benefits in terms of reactivity, which will surely lead to an increase in the mechanical properties of the cement. Therefore, the pyrolysis process must seek a yield of obtaining biochar at any temperature, since this work has shown that temperature is not an important factor in the final by-product (biochar) for its reactivity in the pozzolanic reaction. That the highest yield of biochar should be sought in the pyrolysis process to obtain a greater quantity of product that can be used as a replacement for cement in the manufacture of low-performance inputs. This behavior has been observed in the 90 and 180 days of tests, observing a greater presence of these gels, both C–H–S and C-A-S-H, which shows that the rice husk biochar is reactive with lime pastes, making its use promising as a replacement for cement. It is evident in Figure 5b and c much more pronounced peaks in the range of 40–150 °C, thus demonstrating that there is formation of these gels as has been reported in the literature [44, 60, 62, 66].

### 3.4 Determination of functional groups

**Figure 6:**
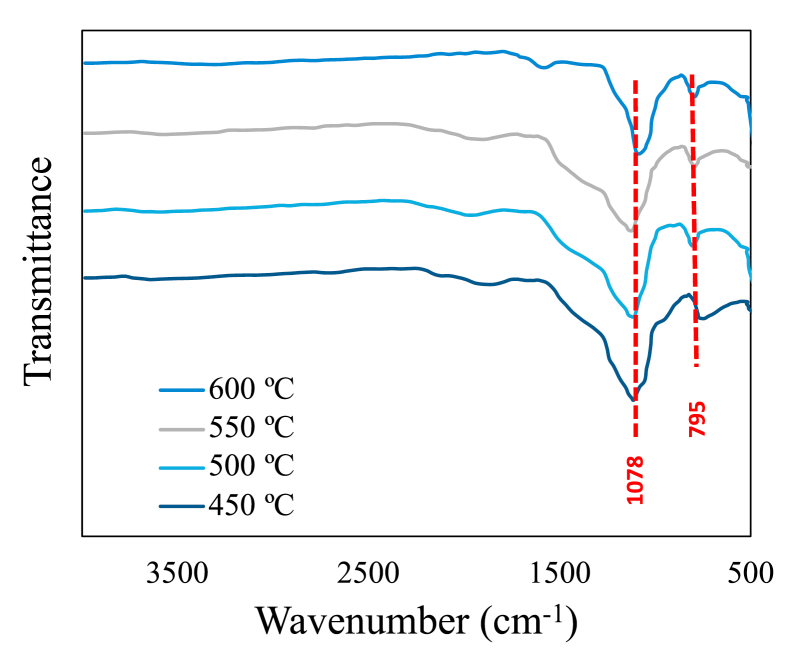
Fourier Transform Infra-red spectroscopy (FTIR) spectra for biochars.

In Figure 6, the FTIR analyzes performed on all biochar are shown. It is evident with these tests that the biochars do not present contents of pectin, hemicellulose, which are associated with vibrations of –OH group at 3800 cm^−1^ [67, 68]. This absence may be due to dehydration of the rice husk due to the pyrolysis temperatures used [69]. All FTIR spectra analyzes reveal the presence of two peaks in common in different ranges, between 1000 and 1100 cm^−1^ and among 700 and 800 cm^−1^. Both peaks can be attributed to the presence of Si–O–Si structures with stretching and curvature vibrations [69, 70, 71]. Therefore, these peaks are related to the mineral composition of Si present in the rice husk.

**Figure 7:**
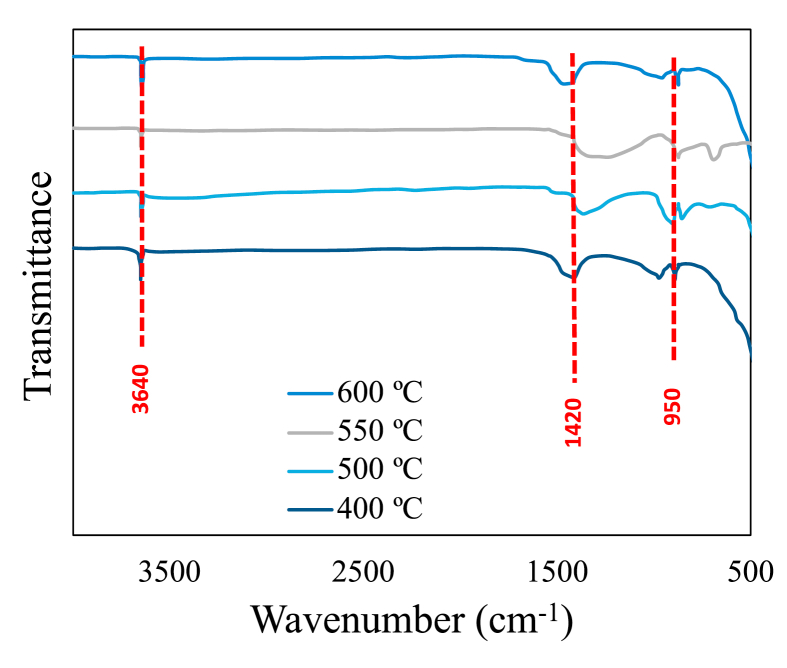
Fourier Transform Infra-red spectroscopy (FTIR) spectra for 25% replacement at 90 days. a) 450 °C, b) 500 °C, c) 550 °C and d) 600 °C.

Figure 7 shows the tests with 25% replacement for all biochars at day 90 of curing. Two peaks between 870 and 950 cm^−1^ are observed, which are related to the hydration of portland cement and the primary production of C–H–S gels [72]. According to other authors these peaks are also related to Si–O vibrations [73, 74]. These two peaks are related to the production of C-A-S-H and C–S–H gels [72]. For the peak shown in the band 1420 cm^−1^ related to C–O stretching, they are characteristic bands of CO_3_^2-^ [73, 74]. It is then observed with these analyzes that at day 90 a pozzolanic reaction of all biochars tested in this study has already occurred; with the formation of C-A-S-H and C–S–H gels, which improve the mechanical characteristics of the cement and that with the TGA analysis of day 180 (Figure 1) the occurrence of these is much more evidenced. Likewise, the peak defined in the 3641 cm^−1^ band can be attributed to the OH band of calcium hydroxide. It is evident that the intensity of all samples is similar and with this it is corroborated that the pyrolysis process to obtain biochar from rice husk for use as a replacement for cement, should seek its maximum production and not focus on the temperature of the process as an important factor.

## 4 Conclusions

The use of biochar from rice husk pyrolysis has proven to be a suitable material for the replacement of cement in the construction industry. All the biochar obtained at different temperatures show high reactivity in all the replaced percentages, but it is the 20 and 25% that show the best performance on day 180. From day 28 to day 180 where the tests have stopped, according to the TGA analyzes, it was possible to show the pozzolanic reactions that were being produced in all the replacements and biochars tests. This demonstrates the feasibility of being used both in the production of Portland as well as a replacement for the manufacture of low-performance inputs. It has been observed that from the first day of curing the portlandite reaction occurs and the formation of C–S–H gels begins, but it is on day 180 that a more pronounced peak is evidenced in the range between 50 and 150 °C, which is associated with this type of gels. Likewise, the reaction of portlandite has shown that in the first 28 days the peak increases between 350 and 450 °C and that then a decrease is noticed on day 56 of curing. Similarly, according to the FITR tests, the formation of both C-A-S-H and C–S–H gels is observed, which contribute to better mechanical behavior of the biochars as a replacement for cement for the manufacture of low-performance inputs. These results show that the biochar from the pyrolysis of rice husk is suitable as a replacement for cement, mainly due to its lignin content.

Based on these results, it is expected to continue advancing in the application of this biochar and others from different biomasses, as well as the mixture of these biochar, in order to find a maximum use of this by-product of pyrolysis. According to the results of this work, it is estimated that 20% of cement can be replaced by this material, reducing manufacturing costs, which in the case of Colombia 1 kg of cement costs approximately US $ 0.13. Therefore, when manufacturing a 10 kg structure, a traditional structure would cost US $ 1.3 while replacing the cement with rice husk biochar the price would be US $ 1.04. Likewise, the costs of exploitation and/p treatment of biochar for another application would be reduced. Contributing in this way to the circular economy of various industries, such as agriculture, cement, energy and construction.

## Declarations

### Author contribution statement

Luisa F. Morales & Katherine Herrera: Performed the experiments; Analyzed and interpreted the data.

Julián E. López: Performed the experiments; Analyzed and interpreted the data; Wrote the paper.

Juan F. Saldarriaga: Conceived and designed the experiments; Performed the experiments; Analyzed and interpreted the data; Contributed reagents, materials, analysis tools or data; Wrote the paper.

### Funding statement

This work was supported by the Department of Civil and Environmental Engineering at 10.13039/501100006394Universidad de los Andes, and by the call for proposals CI-0120: "Publish your new knowledge or expose your new creations" from the Office Vice President for Research and Creation at Universidad de los Andes.

### Data availability statement

Data included in article/supp. material/referenced in article.

### Declaration of interests statement

The authors declare no conflict of interest.

### Additional information

No additional information is available for this paper.
